# Hair quantitative PCR outperforms blood analysis for detecting *Leishmania* DNA in dogs: a non-invasive tool for clinical states of infection

**DOI:** 10.1186/s13071-026-07449-3

**Published:** 2026-05-27

**Authors:** Hafiz Muhammad Safwan, M. Magdalena Alcover, Andrea Murillo-Picco, Marta Baxarias, Roser Fisa, Laura Ordeix, Laia Solano-Gallego

**Affiliations:** 1 Departament de Medicina i Cirurgia Animals, Facultat de Veterinària, Universitat Autònoma de Barcelona, Cerdanyola del vallès, Spain; 2 Departament de Biologia, Sanitat i Medi Ambient, Facultat de Farmàcia i Ciències de l’ Alimentació, Universitat de Barcelona, Barcelona, Spain; 3 Servei de Dermatologia, Hospital Clínic Veterinari, Universitat Autònoma de Barcelona, Cerdanyola del vallès, Spain

**Keywords:** Canine leishmaniosis, ELISA, IFN-γ, Molecular diagnosis, Subclinical infection

## Abstract

**Background:**

Canine leishmaniosis, caused by *Leishmania infantum,* is a significant zoonotic disease threatening both canine and human health worldwide. Early and accurate diagnosis is crucial for effective treatment and control. The use of hair samples for molecular diagnosis of *L. infantum* infection in dogs is a relatively novel approach and has not yet been extensively studied. Despite recent advances in blood-based polymerase chain reaction (PCR), noninvasive alternatives like hair quantitative PCR (qPCR) remain underexplored for different clinical states of infection. This study evaluates the utility of hair samples for qPCR diagnosis compared with traditional blood-based methods in different clinical states of infection.

**Methods:**

A total of 134 dogs from various regions of Spain, classified into healthy seronegative (*n* = 14), healthy seropositive (*n* = 59), or clinically sick (*n* = 61) were studied. Most sick dogs were in LeishVet stage IIa. Hair ear (*n* = 97) or neck (*n* = 37) and blood samples (*n* = 134) were collected. Diagnostic methods included quantitative in-house enzyme-linked immunosorbent assay (ELISA), endpoint ELISA, interferon gamma (IFN-γ) release whole blood assay, and hair and blood qPCR for *Leishmania* spp.

**Results:**

Hair qPCR showed significantly (*P* < 0.001) higher sensitivity in sick dogs (74%) compared with blood qPCR (36%) while no differences were found in healthy seropositive dogs (*P* = 0.593). IFN-γ production was not associated with hair qPCR positivity in either healthy seropositive or sick dogs. However, medium/high ELISA seropositivity was associated with substantially increased qPCR positivity in both blood (*P* = 0.002) and hair (*P* < 0.001). Sick dogs exhibited significantly higher antibody levels (*P* < 0.001), while healthy seropositive dogs showed stronger IFN-γ responses (*P* = 0.027).

**Conclusions:**

Hair qPCR is a sensitive, non-invasive diagnostic tool for detecting *Leishmania* DNA in sick dogs.

**Graphical Abstract:**

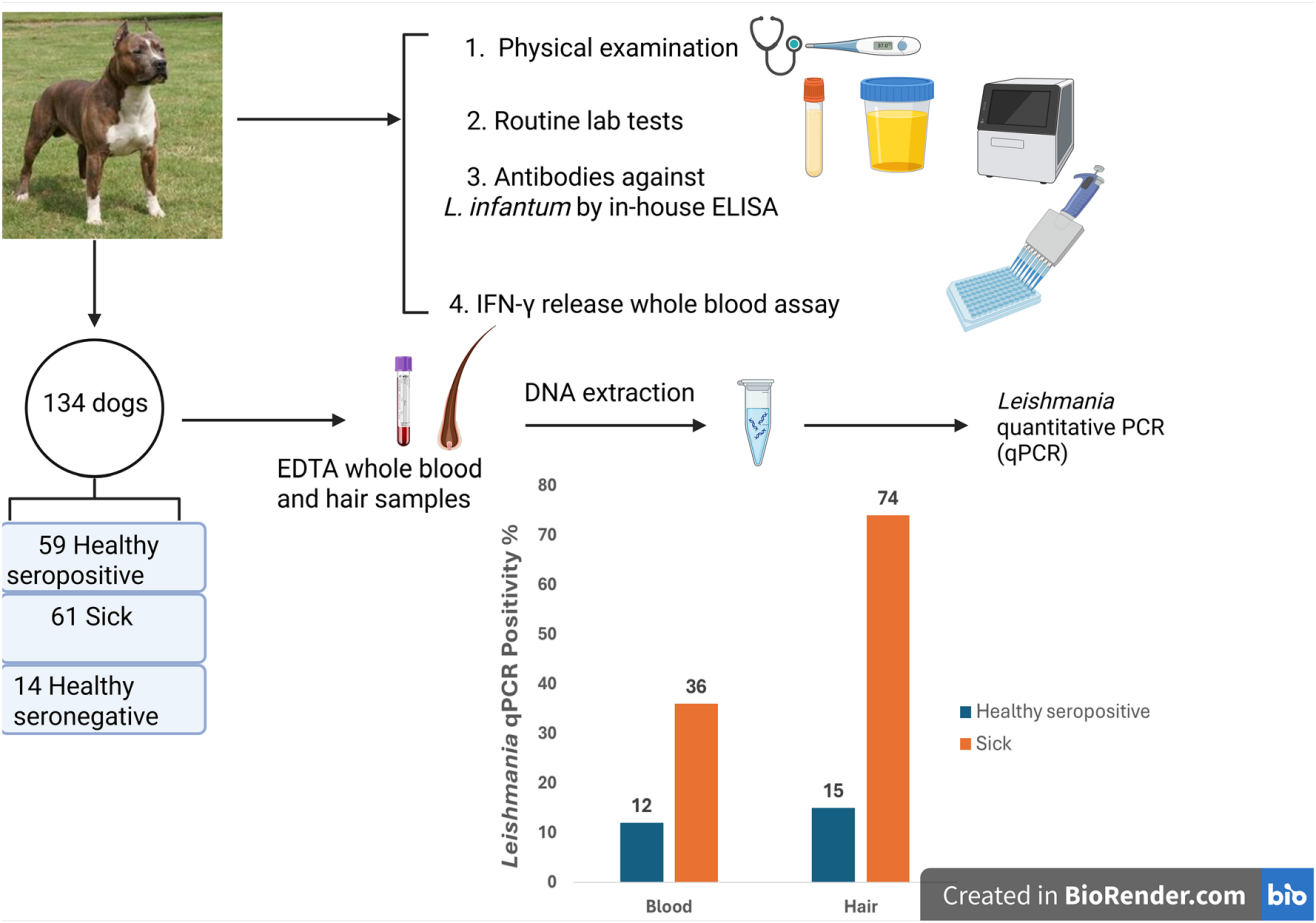

**Supplementary Information:**

The online version contains supplementary material available at 10.1186/s13071-026-07449-3.

## Background

Canine leishmaniosis (CanL), caused by the protozoan parasite *Leishmania infantum*, is a serious threat to both canine and human health in endemic areas worldwide [1, 2]. Clinical manifestations range from subclinical infection to severe systemic illness [3]. Cutaneous signs (e.g., exfoliative, erosive–ulcerative, nodular, papular, or pustular dermatitis and onychogryphosis) and systemic abnormalities (e.g., generalized lymphadenomegaly, splenomegaly, renal dysfunction) are hallmark features. Ocular signs such as blepharitis, conjunctivitis, keratoconjunctivitis, anterior uveitis, and endophthalmitis are also frequent. The biochemistry profile often reveals hyperglobulinemia (polyclonal beta and/or gammopathy), hypoalbuminemia, and proteinuria. Hematological abnormalities may include mild-to-moderate nonregenerative anemia, leukocytosis or leukopenia, thrombocytopathy, and thrombocytopenia, with potential impairments in hemostasis. Other less common clinical problems can involve mucocutaneous ulcerations, epistaxis, polyarthritis, lameness, myositis, vasculitis, arterial thromboembolism, and neurological disorders [3].

Clinical staging of CanL is generally categorized into four stages, on the basis of the severity of clinical signs and laboratory abnormalities [3]. In stage I, sick dogs may exhibit mild clinical signs, with only localized lymphadenomegaly and mild skin lesions, such as papular dermatitis [4]. Laboratory findings are usually normal. Stage II involves moderate disease with more pronounced clinical signs, including generalized lymphadenomegaly, weight loss, generalized or multifocal cutaneous signs, and common laboratory abnormalities such as mild normocytic normochromic nonregenerative anemia, hyperglobulinemia, or mild proteinuria. Stage III is characterized by clinical signs and laboratory abnormalities compatible with CanL and evidence of kidney disease in all cases. In stage IV, the disease is very advanced and dogs have severe kidney disease. Other severe complications, such as systemic vasculitis or organ failure, are common at this stage, significantly impacting the prognosis [2, 3].

Early and accurate diagnosis of this infection and disease is crucial for effective treatment and control measures [5, 6]. The diagnosis of *L. infantum* infection, particularly in dogs, involves various diagnostic techniques that provide differing levels of sensitivity and specificity, depending on the clinical stage and state of infection. One of the most common diagnostic approaches is serology, which detects antibodies produced in response to *L. infantum* antigen [7]. Among the serological methods, the immunofluorescence antibody test (IFAT) and enzyme-linked immunosorbent assay (ELISA) are widely used owing to their effectiveness in identifying both subclinical and clinical infections. These tests can measure antibody levels, which correlate with disease progression, making them valuable tools for screening large populations in endemic areas [8].

In addition to serology, cytology and histopathology remain important diagnostic tools, particularly when clinical signs are present. In these methods, tissue samples (e.g., lymph nodes, bone marrow, or skin) are examined under a microscope for the presence of *Leishmania* amastigotes [9]. Although cytology is less sensitive than serological diagnostic techniques, it provides immediate confirmation of infection when positive. Histopathology, which involves staining tissue samples with routine stains like hematoxylin and eosin, is especially valuable when biopsies are taken from skin lesions or organs where the parasite load is expected to be high. The main limitation of these techniques is that they require significant expertise, are invasive, and may yield false negatives when the parasitic load is low. In these cases, *Leishmania* immunohistochemistry is recommended [9].

Molecular diagnostic techniques, particularly quantitative PCR (qPCR), have significantly advanced the diagnosis of *L. infantum* in both clinical and research settings. These methods offer superior sensitivity and specificity compared with traditional diagnostics, as well as the ability to quantify parasite load and monitor treatment response [5, 6]. Moreover, qPCR can differentiate between *Leishmania* species and strains, contributing valuable epidemiological data for disease surveillance in endemic regions [7].

The use of noninvasive samples such as hair for molecular diagnosis of CanL through qPCR is increasing. The use of qPCR in noninvasive samples has marked substantial progress in *Leishmania* diagnoses, with hair emerging as a promising specimen type [10–13]. Previous studies have demonstrated that *L. infantum* DNA can be detected in canine hair using PCR-based methods, supporting the feasibility of this matrix for molecular diagnosis, although reported sensitivities have varied depending on the target sequence and sampling strategy [11, 13]. Hair samples have several distinct advantages: they are easy to collect without causing distress to the animal, they can be stored at room temperature without special preservation requirements and they contain detectable parasite DNA even months after collection [10]. Recent research has shown that *L. infantum* kinetoplast DNA (kDNA) is stable in hair follicles and shafts owing to the presence of the cuticle and keratin protein that protects the DNA from degradation [10]. This stability makes hair qPCR especially useful for field studies in places where immediate sample processing is not possible. These benefits have made hair qPCR an increasingly significant method in both individual case management [14] and large-scale epidemiological surveillance of CanL [15].

Molecular diagnostic approaches have shown great potential for detecting CanL. The kDNA-based qPCR assay used in this study is one of the most sensitive and widely validated molecular methods for detecting *Leishmania* DNA in dogs in the Mediterranean region. The high copy number of kDNA makes this target particularly suitable for samples with low parasite burden [16–18]. In another study, qPCR is used for detecting *Leishmania* spp. DNA in the peripheral blood of seropositive dogs, indicating its efficacy as a diagnostic tool when compared with established serological approaches [19]. Their findings confirmed qPCR as a useful method for confirming infection in dogs who tested positive for serological screening, emphasizing the complementary significance of molecular approaches in disease surveillance programs.

The application of hair samples for molecular diagnosis of *L. infantum* is not limited to domestic animals but also extends to wild animals. Another study [12] developed a specific qPCR technique to detect *L. infantum* DNA in wild Leporidae, focusing on hair samples as a noninvasive diagnostic approach. Their findings showed that hair could be a viable sample type for parasite molecular identification, with advantages over typical invasive sampling methods such as spleen or skin biopsies. This groundbreaking study on hair-based qPCR detection in wildlife demonstrated the potential for developing practical field surveillance techniques that minimize handling stress while maintaining diagnostic sensitivity, laying the groundwork for noninvasive monitoring of *L. infantum* in potential wildlife reservoirs.

Understanding the performance of qPCR from hair samples for the diagnosis of *Leishmania* infection at different states of infection can have profound implications for clinical management and monitoring. There are very few studies available so far for the molecular diagnosis of *Leishmania* through the detection of DNA using hair samples in dogs [10, 11, 13, 20]. Therefore, this study aimed to determine the usefulness of qPCR from hair and blood samples in diagnosing different states of *L. infantum* infection in dogs.

## Mthods

### Dogs and sampling

A total of 134 client-owned dogs from different regions of Spain were enrolled in the study. These dogs were classified into healthy seropositive (*n* = 59), healthy seronegative (*n* = 14), and sick dogs (*n* = 61) on the basis of clinical examination, routine laboratory tests, ELISA test for antibodies for *L. infantum* antigen, and interferon gamma (IFN-γ) release whole blood assay (WBA) for the detection of IFN-γ against *L. infantum* soluble antigen (LSA). Signalment data were recorded. Sample collection took place between September 2020 and March 2024 across various regions in Spain. Hair samples were obtained using disinfected (30% bleach and soap in distilled water before each of use) Halsted mosquito forceps and were stored in Eppendorf tubes. Apart from disinfection of the forceps, no standardized cleaning or additional pretreatment of the hair samples was performed at the time of collection.

Blood samples were taken from jugular or cephalic venipuncture, which were then immediately transferred into various tubes: ethylenediaminetetraacetic acid (EDTA) tubes for *Leishmania* qPCR and complete blood count (CBC) (Spain: XN1000, Sysmex España SL, Sant Just Desvern, Spain; Italy: Advia 2120, Siemens Healthcare SRL, Milano, Italy); plain serum tubes for serum electrophoresis (Spain and Italy: Capillarys 3, Sebia Dubai SA, Dubai, UAE) and biochemistry profile (Italy: AU 5800, Beckman Coulter International SA, Nyon, Switzerland); and heparin tubes for the WBA used to measure canine IFN-γ. Plucked hair samples were obtained from the ear (*n* = 97) in 45 healthy seropositive and 52 sick dogs and from the neck (*n* = 37) in 14 healthy seronegative, 14 healthy seropositive, and 9 sick dogs. Biochemistry profile included urea, creatinine, total proteins, albumin, total globulins, albumin/globulin (A/G) ratio, alkaline phosphatase (ALP), and alanine aminotransferase (ALT). Free catch or cystocentesis methods were used to collect urine samples for urinalysis. The urine-specific gravity, sediment analysis, urinary protein/creatinine ratio (available in Spain from Vitros 5600, Ortho Clinical Diagnostics, New York, NY, USA, and in Italy from Cobas U601, Roche, Buenos Aires, Argentina), and urine strip test (Beckman Coulter International SA, Nyon, Switzerland) were recorded [21].

### Quantitative in-house ELISA for the detection of *L. infantum*-specific antibodies

Serum from each dog was tested using an in-house ELISA as previously described [21]. Briefly, samples were diluted to 1:800 in 1% dry milk-containing phosphate-buffered saline (PBS)-Tween and then incubated for 1 h at 37 °C in *L. infantum* antigen-coated plates (20 µg/mL). Subsequently, the plates underwent three PBS-Tween washes and one PBS-only wash before being incubated for 1 hour at 37 °C with protein A conjugated to horseradish peroxidase (concentration 0.16 ng/µL, peroxidase conjugate protein A; Merck KgaA, Darmstadt, Germany). The plates were then washed again. Substrate buffer (Sigma fast OPD; Merck KgaA, Darmstadt, Germany) and substrate solution (*o*-phenylenediamine dihydrochloride) were added to the plates to develop them. A total of 50 µL of 2.5 M H_2_SO_4_ was used to stop the reaction. An automatic ELISA reader (MB-580 Heales; Shenzhen Huisong Technology Development Co., Ltd., Shenzhen, China) was used to measure absorbance values at 492 nm. Each plate included healthy negative control and confirmed sick high positive control. Samples were tested in duplicates. Results were expressed in ELISA units (EU), set arbitrarily at 100 EU relative to a calibrator. Positivity was classified as high (≥ 300 EU), medium (150–299 EU), or low (25–149 EU) [22]. This in-house ELISA has similar sensitivities and specificities with IFAT [23–26].

### Endpoint ELISA for quantification of *L. infantum*-specific antibodies

A twofold serial dilution ELISA was used to analyze all samples with an ELISA unit value greater than 150 EU by in-house ELISA. Sera twofold dilutions started at 1:800 and continued for 9–11 dilutions. The result was expressed as ELISA units (EU) relative to a calibrator set arbitrarily at 100 EU and an optical density (OD) value of 1 at the 1:800 dilution. The positivity% was calculated as: (Sample OD/Calibrator OD) × 100 × dilution factor) utilizing the mean values of dilutions with an OD near 1 as previously described [22].

### DNA extraction from blood and hair samples

EDTA–blood DNA extraction was carried out with a commercial blood DNA extraction kit (MagMAX CORE Nucleic Acid Purification Kit, Thermo Fisher Scientific Inc., Waltham, MA, USA) following the manufacturer’s instructions using an automated system (King Fisher Flex Purification System, Thermo Fisher Scientific Inc., Waltham, MA, USA). DNA from hair samples was extracted using the High Pure PCR Template Preparation Kit (Roche Applied Science, Mannheim, Germany), following the manufacturer’s protocol.

### *Leishmania* qPCR

After DNA extraction from blood and hair samples, qPCR was performed in a total reaction volume of 10 μL, containing 0.75 μL of forward primer (5′-CTTTTCTGGTCCTCCGGGTAGG-3′), 0.75 μL of reverse primer (5′-CCACCCGGCCCTATTTTACACCAA-3′), 0.5 μL of TaqMan probe (5′-FAM-TTTTCGCAGAACGCCCCTACCCGC-TAMRA-3′), 5 μL of iTaq Supermix with ROX (Bio-Rad), 0.5 μL of PCR-grade sterile water, and 2.5 μL of sample DNA. The final concentration corresponded to 15 pmol of each primer and 5 pmol of probe per reaction.

The qPCR protocol for *Leishmania* DNA detection followed previously described methods [27, 28]. Briefly, amplifications were carried out on a Quant Studio 7 Pro Real-Time PCR System (Applied Biosystems) using a two-step thermal cycling protocol (95 °C and 55 °C) for 45 cycles. All reactions were performed in triplicate, and each run included negative controls, positive controls, and a standard curve. The standard curve was generated using tenfold serial dilutions of *L. infantum* DNA, ranging from 10^5^ to 10^−3^ promastigotes/mL. To minimize false-positive results, particularly those associated with late amplification, samples were only considered positive if amplification was observed in at least two of the three replicates with a quantification cycle (*Ct*) value less than or equal to 37.

### IFN-γ release whole blood stimulation assay

The IFN-γ release WBA was carried out as previously described [21, 29–32]. Heparinized whole blood was diluted at a 1:10 dilution with culture medium, and 300 µL blood was dispensed into wells containing three conditions: medium alone, LSA (10 µg/mL), or concanavalin A (10 µg/mL). Cultures were incubated for 5 days at 37 °C and 5% CO_2_. After 5 days of incubation, samples were centrifuged at 1600 rpm for 10 min. The supernatants were collected and stored at −80 °C until analysis. Sandwich ELISA (DuoSet^®^, R&D Systems, UK) was used to quantify IFN-γ concentrations using a standard curve ranging from 4000 to 62.5 pg/mL. Plates were read at 450 nm and analyzed using a four-parameter logistic curve (MyAssays application). The standard curve was repeated if the *R*^2^ was less than 0.98. All samples were examined in duplicate. Dogs were categorized as IFN-γ producers when antigen-specific IFN-γ levels were ≥ 110 pg/mL [29–32]. WBA was not performed in six healthy seropositive dogs and five sick dogs.

### Statistical analysis

The Shapiro–Wilk test was used to determine whether the quantitative variables had a normal distribution or not. Nonparametric Mann–Whitney *U* test was used to compare numerical data (age, weight, antibody levels, IFN-γ concentrations, and qPCR *Ct* values) between only healthy seropositive and sick dog groups, whereas categorical variables (sex, breed, antibody, qPCR, and IFN-γ results) were evaluated using chi-squared test. Chi-squared test of independence was used to check the association between the ELISA antibodies, ELISA IFN-γ, and the blood/hair qPCR *Leishmania* test. The McNemar test and the Wilcoxon signed-rank test were performed to check the statistical difference in the paired samples. *P*-values less than 0.05 were considered statistically significant. The software JMP (student edition version 18), GraphPad Prism (Version 9, San Diego, CA) and The Jamovi Project (2022) (Version 2.3.8) (Computer Software) were used for statistical analysis.

## Results

### Signalment characteristics and clinical data

The clinical, serological, and molecular results of the healthy seropositive and sick dogs are presented in Tables 1 and 2. The Andalusian hound and Border Collie were the most common breeds in the healthy seronegative dogs with the range of age from 8 months to 10 years. There were 79% females and 21% males in this group.

**Table 1:** Qualitative characteristics and results of different diagnostic tests of healthy seropositive and sick dogs studied

Variable	Total (*n* = 120)	HSP (*n* = 59)	Sick (*n* = 61)	Chi-squared test, *P*-value
	% (95% CI)	% (95% CI)	% (95% CI)	
Breed				
Crossbreed (*n* = 54)	45 (36–54.3)	53 (39–66)	38 (26–51)	*χ*^2^ = 2.67*, df* = 1*, P* = 0.102
Purebreed (*n* = 66)	55 (46–64.2)	47 (34–61)	62 (49–74.3)	
Sex				
Male (*n* = 58)	48 (40–58.5)	48 (36–63)	49 (36.1–62.3)	*χ*^2^ = 0.035*, df* = 1*, P* = 0.850
Female (*n* = 62)	52 (41.1–60.1)	52(37–64)	51 (38–64)	
ELISA interpretation				
High or medium seropositive (*n* = 54)	45 (36–54)	22 (13–35)	78 (65–87)	*χ*^2^ = 30.4*, df* = 1*, P* < 0.001
Low seropositive (*n* = 66)	55 (46–63)	71 (59–81)	29 (18–40)	
LSA IFN-γ production^*^				
Producer (*n* = 55)	50 (41–60)	60 (47–72)	41 (29–54)	*χ*^2^ = 4.90*, df* = 1*, P* = 0.027
Nonproducer (*n* = 54)	50 (40–59)	40 (27–53)	60 (46–71)	
Blood *Leishmania* qPCR				
Positive	24 (17–33)	12 (5–23)^a^	36 (24–49)^b^	*χ*^2^ = 9.59*, df* = 1*, P* = 0.002
Negative	76 (67.1–83.1)	88 (77–95)	64 (51–76)	
Hair *Leishmania* qPCR				
Positive	45 (36–54)	15 (7.2–27)^a^	74 (61–84)^b^	*χ*^2^ = 41.5*, df* = 1, *P* < 0.001
Negative	55 (46–64)	85 (73–93)	26 (16–39)	

*CI* confidence interval, *ELISA* enzyme-linked immunosorbent assay, *HSP* healthy seropositive dogs, *IFN-γ* interferon gamma, *LSA Leishmania infantum* soluble antigen, *qPCR* real-time quantitative PCR

^a^There was no statistically significant difference between the qPCR results of blood and hair samples from paired healthy seropositive dogs (McNemar test, *χ*^2^ = 0.286, *df* = 1, *P* = 0.593)

^b^There was a statistically significant difference between hair and blood qPCR results of paired samples from sick dogs (McNemar test, *χ*^2^ = 18.2, *df* = 1, *P* < 0.001)

^*^Interferon gamma results from six healthy seropositive dogs and five sick dogs were not performed

**Table 2:** Quantitative characteristics and results of the different diagnostic tests of healthy seropositive and sick dogs studied

Variable	Total (*n* = 120)	HSP (*n* = 59)	Sick (*n* = 61)	Mann–Whitney *U* test, *P*-value
	Median (min–max)	Median (min–max)	Median (min–max)	
Age (years)	5 (1–14)	4 (1–14)	5 (1–12)	*U* = 1318*, P* = 0.022
Endpoint ELISA (EU)	417.4 (57–8501)	193 (57–1181)	1094 (87–8501)	*U* = 650*, P* < 0.001
IFN-γ (pg/mL)				
LSA	137 (0–10,220)	226 (0–10,220)	78 (0–5994)	*U* = 1120*, P* = 0.026
Con-A	5669 (573–38,260)	5986 (573–26,300)	5553 (804–38,260)	*U* = 1242*, P* = 0.143
Blood *Leishmania* qPCR (*Ct* values)	31.0 (23.1–36.7)	36.7 (30.4–34.8)^a^	31.0 (23.1–36.5)^b^	*U* = 160*, P* = 0.009
Hair *Leishmania* qPCR (*Ct* values)	33.05 (24–36.9)	36 (32–36.9)^a^	32.6 (24–36.5)^b^	*U* = 117*, P* ≤ 0.001

*Con-A* concanavalin-A, *Ct* threshold cycle, *df* degrees of freedom, *ELISA* enzyme-linked immunosorbent assay, *EU* enzyme-linked immunosorbent assay units, *HSP* healthy seropositive dogs, *IFN-γ* interferon gamma, *LSA Leishmania infantum* soluble antigen, *Max* maximum, *Min* minimum, *pg/mL* picogram/milliliter, *qPCR* real-time quantitative PCR

^a^Wilcoxon signed-rank test showed no statistically significant difference between blood and hair qPCR *Ct* values of paired samples in healthy seropositive dogs (Wilcoxon signed-rank test, *Z* = 16.0,* P* = 0.313)

^b^Wilcoxon signed-rank test showed no statistically significant difference between blood and hair qPCR *Ct* values of paired samples in sick dogs (Wilcoxon signed-rank test, *Z* = 416, *P* = 0.197)

Sex distribution was remarkably balanced across healthy seropositive and sick dogs, with males constituting 48% of healthy seropositive and 49% of sick dogs (Table 1). The age range of infected dogs was 1–14 years. Both purebred (55%) and crossbred dogs (45%) were represented in healthy seropositive and sick groups. In these groups, purebred dogs were the majority (20 breeds were recorded in total), with American Staffordshire terrier, German shepherd, and Border Collie being the most popular breeds. No statistically significant difference was observed in breed distribution between healthy seropositive and sick dogs (Table 1). Sick dogs were further classified as LeishVet stage IIa (*n* = 35), IIb (*n* = 6), III (*n* = 9), and IV (*n* = 1). Ten dogs were categorized in stage II owing to missing urinary data.

### ELISA antibodies and IFN-γ release whole blood assay

The serological analysis across clinical groups indicated that sick dogs had a larger percentage of medium-to-high antibody levels (70%) than healthy seropositive dogs (20%) (chi-squared test, *χ*^2^ = 30.4*, df* = 1*, P* < 0.001; Table 1). Endpoint ELISA findings showed a similar pattern, with sick dogs having significantly higher median antibody levels (1094 EU) than healthy seropositive dogs (193 EU) (Mann–Whitney *U* test, *U* = 650, *P* < 0.001), as presented in Table 2. Healthy seropositive dogs were significantly more IFN-γ producers (60%) than sick dogs (41%) (chi-squared test, *χ*^2^ = 4.90*, df* = 1*, P* < 0.027; Table 1). Furthermore, healthy seropositive dogs produced more LSA-stimulated IFN-γ (median 226 pg/mL) than sick dogs (median 78 pg/mL), (Mann–Whitney *U* test, *U* = 1120, *P* = 0.026; Table 2). All healthy seronegative dogs were non-IFN-γ producers.

### *Leishmania* qPCR

Hair qPCR showed remarkable diagnostic power in detecting *Leishmania* infection, with considerably higher positivity rates in clinically sick dogs (74%) compared with healthy seropositive dogs (15%) (chi-squared test, *χ*^2^ = 41.5*, df* = 1*, P* < 0.001; Table 1). Quantitative analysis in Table 2 showed that sick dogs had significantly lower median *Ct* values than healthy seropositive dogs (Mann–Whitney *U* test, *U* = 117*, P* < 0.001), showing higher parasite burdens in hair samples from clinically affected animals.

Table 3 presents the qPCR positivity percentage in blood and hair samples from healthy seronegative, healthy seropositive, and clinically sick dogs, with variations depending on the location of hair collection. Among healthy seropositive dogs, ear hair was more frequently positive (20%) than neck hair (0%) (chi-squared test, *χ*^2^ = 3.30*, df* = 1*, P* = 0.069*)* and among sick dogs, a much higher positivity rate (86.5%) than neck-hair samples (0%) was observed (chi-squared test, *χ*^2^ = 29.69*, df* = 1*, P* < 0.001). *Leishmania* DNA was detected in ear hair (55.7%) but all the neck-hair samples were negative to hair *Leishmania* qPCR across all dog groups (chi-squared test, *χ*^2^ = 23.28*, df* = *1, P* < 0.001). Only one healthy seropositive dog out of 59 dogs (2%, 95% confidence interval [CI] 0.04–9.09) had both positive blood and hair qPCR results. In contrast, 19 sick dogs out of 61 dogs (31.2%, 95% CI 20–44.2) showed simultaneous positive results in both blood and hair samples. Among these, most dogs were classified as LeishVet stage IIa (*n* = 12), followed by stage III (*n* = 7).

**Table 3:** qPCR positivity percentage in blood and hair samples by anatomical location of hair sampling and health status

Anatomical location (number of dogs)	Dog group (number of dogs)	Blood qPCR positive (%) (95% CI)	Hair PCR positive (%) (95% CI)
Ear hair (*n* = 97)	Healthy seropositive (*n* = 45)	15.5 (6.5–29.4)	20 (9.5–35)^a^
	Sick dogs (*n* = 52)	40.38 (27.01–55)	86.5 (74.2–94.4)^b^
Neck hair (*n* = 37)	Healthy seronegative (*n* = 14)	0	0
	Healthy seropositive (*n* = 14)	0	0^a^
	Sick dogs (*n* = 9)	11 (0.23–41.3)	0^b^

*qPCR* real-time quantitative PCR, *CI* confidence interval

^a^chi-squared test, *χ*^2^ = 3.30*, df* = 1*, P* = 0.069

^b^chi-squared test, *χ*^2^ = 29.69*, df* = 1*, P* < 0.001

All healthy seronegative dogs were negative by qPCR for both hair and blood samples.

### Association between immunological and molecular parameters

There was no significant association between IFN-γ production and hair qPCR positivity when evaluated according to health status. Among healthy seropositive dogs (*n* = 53), producers and nonproducers showed similar qPCR positivity rates (chi-squared test: *χ*^2^ = 0.0412, *df* = 1, *P* = 0.521; odds ratio [OR] = 1.759, 95% CI: 0.308–10.04). Similarly, in clinically sick dogs (*n* = 56), no significant association was identified (chi-squared test, *χ*^2^ = 0.265, *df* = 1, *P* = 0.607; OR = 0.731, 95% CI: 0.22–2.41). When all dogs were analyzed together, IFN-γ production was not significantly linked with hair qPCR positivity (chi-squared test, *χ*^2^ = 1.544, *df* = 1, *P* = 0.214; OR = 0.61, 95% CI: 0.29–1.32).

Interestingly, a significant association was found between ELISA seropositivity and qPCR positivity in both blood and hair samples. In blood, dogs with medium/high ELISA positivity were more likely to test qPCR positive compared with those with low ELISA positivity (chi-squared test: *χ*^2^ = 12.47, *df* = 1, *P* < 0.001; OR = 5.60, 95% CI: 2.19–14.30). Similarly, medium/high ELISA positivity was strongly associated with qPCR positivity in hair samples (chi-squared test: *χ*^2^ = 10.62, *df* = 1, *P* < 0.001; OR = 3.74, 95% CI: 1.78–7.85).

## Discussion

The current study reveals significant differences between clinically sick and healthy seropositive dogs across various diagnostic measures, including hair qPCR, highlighting the diagnostic value of hair qPCR in CanL. Our findings provide important new insights into the relationship between parasite detection techniques, antibody levels, and cell-mediated immune responses during different clinical states of *L. infantum* infection. The most striking result of the present study is that hair qPCR demonstrates a remarkable sensitivity in detecting *L. infantum* infection in clinically compromised dogs, compared with healthy seropositive dogs. In another study conducted in Brazil [11], the authors reported that 80% of infected dogs tested positive for *L. infantum* DNA in hair using conventional PCR, in agreement with the present study. That study also found that hair conventional PCR positivity was significantly associated with infectivity to sand flies, as confirmed by xenodiagnoses in 67% of cases [11]. The detection of *L. infantum* DNA in infected dog hair has been identified as a potential method for determining infectiousness to *Lutzomyia longipalpis* [11]. In a recently published study conducted in Brazil, hair was used as a noninvasive matrix for the detection of *Leishmania* by nested ITS1 PCR [13]. The authors reported a sensitivity of 59.1%, for hair samples, which outperformed blood nested ITS1 PCR [13] but was lower than the sensitivity observed in the present study.

*Ct* values were significantly lower in sick dogs compared with healthy seropositive dogs, suggesting that parasite load in hair correlates with disease progression [33]. The superior performance of qPCR on hair compared with blood qPCR represents a significant advance in noninvasive diagnosis of CanL. To our knowledge, this study is first study to clearly demonstrate a statistically significantly higher sensitivity of hair qPCR compared with blood qPCR, in clinically sick dogs. This is something different from a previous study conducted in Spain [10], which reported almost similar detection rates between hair and blood with qPCR (69% and 62%, respectively). However, clinical staging of the dogs was not specified, which could explain the observed differences with the study presented herein.

qPCR positivity was observed exclusively in ear-hair samples, whereas none of neck-hair samples tested positive. However, these findings should be interpreted cautiously because neck-hair samples were few and sampling was unpaired, limiting the validity of anatomical comparisons. Such imbalance prevents definitive conclusions regarding site-specific diagnostic performance. Previous studies have reported heterogeneous parasite distribution across skin regions, with higher detection rates in ear and nasal skin [34]. Although our results align with this pattern, the present dataset does not allow confirmation of differential sensitivity between anatomical sites. Future studies employing paired, multi-site sampling within the same dogs and larger sample sizes are needed to reliably assess site-specific qPCR sensitivity.

To determine the presence of *Leishmania * DNA, the method by which the hair sampling is collected matters significantly [10]. In our study, hair samples were taken by plucking with the help of disinfected Halsted tweezers from the ear in 72% of dogs and from the neck in 28% of dogs. Plucked hair is a more informative sample, serving as a mini-biopsy by containing both shaft and root-associated tissue [14]. Cut hair, however, lacks follicular substance and may have lower diagnostic sensitivity. Therefore, plucking is most likely to increase the detection of parasite DNA in hair-based diagnostics. Another crucial aspect of our study is that we collected hair samples from clinically healthy skin areas instead of from any area with clinical skin lesions. This important distinction validates hair qPCR as a highly effective diagnostic technique and suggests its potential use in distinguishing between healthy but subclinical infections and active disease.

In addition, intrinsic hair characteristics such as thickness and pigmentation can influence PCR-based detection [35]. Thicker hair may contain more keratin, potentially hindering DNA extraction if not adequately digested. Pigmentation due to melanin, a known PCR inhibitor, can interfere with polymerase activity and reduce amplification efficiency [35]. These factors highlight the importance of accounting for hair properties when optimizing diagnostic protocols. Moreover, environmental contaminants such as dust or soil particles can introduce PCR inhibitors like humic acids, which impair polymerase function and may lead to reduced sensitivity or false-negative results [36]. This risk reinforces the need for thorough sample cleaning and pretreatment to ensure accurate *Leishmania* DNA detection.

In this study, no standardized cleaning or chemical pretreatment of the hair samples was performed before hair collection, apart from disinfecting the sampling forceps. This represents a methodological limitation because hair contains compounds such as keratin and melanin that may interfere with PCR amplification. However, the extraction protocol used includes an inhibitor removal step, which helps to reduce the effect of these substances during purification, but it may not fully compensate for the lack of external pretreatment. Future work should incorporate a standardized precleaning procedure combined with inhibitor-removal steps during DNA extraction to enhance DNA recovery and minimize the risk of PCR inhibition. Such refinements are expected to further strengthen the diagnostic performance of hair-based sampling in subsequent studies.

We also recognize that using different DNA extraction kits for blood and hair may influence matrix-specific recovery of *Leishmania* DNA. The High Pure PCR Template Preparation Kit was used for hair samples, and to our knowledge, this was the first study applying this kit to canine hair samples. Previous studies have used the High Pure system or similar silica-based methods primarily for blood or tissue samples when performing PCR detection of *Leishmania* spp., showing reliable nucleic acid recovery and compatibility with downstream amplification [6, 17, 37]. Likewise, magnetic bead-based kits such as MagMAX™ CORE are widely used for whole blood because they provide high yield and low inhibitor carryover [38]. Our approach aimed to use extraction procedures appropriate for each matrix to avoid artificially lowering DNA yield. However, inherent differences in sample composition and extraction efficiency between matrices are known to contribute to variation in sensitivity in molecular studies [37, 38]. Comparative studies using noninvasive samples such as hair further highlight the methodological challenges association with achieving consistent sensitivity across different biological matrices [13].

Blood qPCR showed significantly lower discriminatory power than hair qPCR, despite detecting statistically significant differences between clinical groups. Differences in positivity rates and *Ct* values between sick and healthy seropositive dogs were limited, indicating that blood is not an optimal sample for quantitative assessment of parasite burden. These findings are consistent with previous studies showing that skin parasite burden is higher than blood parasite burden and correlates with parasitemia [39] as well as reports indicating that blood parasitemia does not consistently correlate with clinical signs in CanL [32, 40], since parasites may preferentially accumulate in other tissues [41]. Although it is widely accepted that peripheral blood is not the most sensitive sample for detecting *Leishmania*, particularly in clinically healthy seropositive dogs with low or intermittent parasitemia [21, 39, 42–44], blood qPCR was included in this study as a molecular sample commonly used in routine clinical practice and epidemiological surveys. While lymph node aspirates or skin biopsies provide higher sensitivity [13], their invasive nature limits their applicability.

While additional targets such as internal transcribed spacer 1 (ITS1) may improve molecular confirmation or species discrimination, their lower copy number compared with kDNA can substantially reduce analytical sensitivity and increase the risk of false-negative results, particularly in samples with low parasite burden, as demonstrated in comparative studies in dogs [13, 16, 18]. In the present study, all dogs were clinically and immunologically characterized, and all healthy seronegative dogs were consistently negative by both blood and hair qPCR, supporting the high specificity of our kDNA-based assay [10, 45]. Nevertheless, we acknowledge that the inclusion of an additional molecular target would strengthen confirmation. Furthermore, it is important to highlight that in a recent study evaluation hair samples from dogs in Brazil using nested ITS1 PCR and sequencing confirmed the presence of *L. infantum* DNA but also revealed co-amplification of non-*Leishmania* trypanosomatids in a subset of samples, underscoring potential specificity limitations associated with this target [13]. Therefore, these findings highlight the need for further refinement and validation of molecular methodologies to ensure both high sensitivity and specificity in CanL diagnosis [43].

The direct proportion between antibody levels and hair qPCR positivity shows that both measurements reflect disease development [21, 22]. The observed association suggests that seropositivity is a reliable indicator of infection confirmed by PCR in both blood and hair. Interestingly, the strength of association was higher for hair qPCR than for blood qPCR, highlighting the potential of hair as a noninvasive sample for molecular detection that correlates well with serological status. While antibody production shows the humoral immune response to infection, a positive hair qPCR suggests that parasites have spread throughout the hair, including hair follicles distant from skin lesions [13, 14]. It has been established that there is a link between serological status and parasite distribution, with evidence showing that dogs with greater antibody levels often exhibit broader parasite dissemination [29, 32, 46].

Healthy seropositive dogs had considerably greater levels of LSA-stimulated IFN-γ compared with sick dogs. This finding is particularly significant since it emphasizes the importance of cell-mediated immunity in controlling *L. infantum* infection [29–31, 47–50]. The lower IFN-γ production observed in sick dogs with high parasite loads, as indicated by hair qPCR and high antibody levels, agrees with the findings of a previous study [32]. However, the findings of our study indicate that IFN-γ production does not affect the detection of *Leishmania* DNA in hair samples, regardless of the animal’s clinical condition. The nonsignificant odds ratios show that IFN-γ response did not predict molecular detection in this population.

Although this study provides useful insight into diagnosing CanL, some limitations should be acknowledged. The sample size is limited regarding anatomical site. The effect of seasonality has not been considered. To precisely determine the sensitivity of the qPCR approach for the diagnosis of CanL, research utilizing hair samples from more dogs from diverse endemic areas with different states of infection is required in the future. We also recognize that validation of new diagnostic approaches ideally involves comparison with gold-standard methods. However, in CanL, no single universally accepted gold standard exists across all clinical states of infection or different degree of disease severity [51–53]. Thus, the comparison of other tissue samples as lymph nodes or bone marrow aspirates with hair samples would be of great value in future studies. Accordingly, hair qPCR is not proposed as a replacement for established diagnostic methods but as a complementary, noninvasive tool evaluated within a multimodal diagnostic framework that includes clinical evaluation, serology, immunology, and molecular testing.

## Conclusions

Hair qPCR can be a transformative tool for the diagnosis of clinical leishmaniosis, combining high sensitivity with noninvasiveness, while blood qPCR is less sensitive.

## Supplementary Information

Additional file 1.

## Data Availability

All data supporting the main conclusions are available in the manuscript and in Additional file 1 (Supplementary Dataset S1: signalment, clinical data, and molecular and immunological results).

## Abbreviations

ALP: Alkaline phosphatase
ALT: Alanine aminotransferase
CBC: Complete blood count
CI: Confidence interval
CO_2_: Carbon dioxide
DNA: Deoxyribonucleic acid
EDTA: Ethylenediaminetetraacetic acid
ELISA: Enzyme-linked immunosorbent assay
EU: enzyme-linked immunosorbent assay units
IFAT: Immunofluorescence antibody technique
IFN-γ: Interferon gamma
ITS1: Internal transcribed spacer
kDNA: Kinetoplast deoxyribonucleic acid
LSA: *Leishmania infantum* soluble antigen
OD: Optical density
OPD: *O*-phenylenediamine dihydrochloride
OR: Odds ratio
PBS: Phosphate-buffered saline
PCR: Polymerase chain reaction
WBA: Whole blood assay
